# Difference in brain activations during appreciating paintings and photographic analogs

**DOI:** 10.3389/fnhum.2014.00478

**Published:** 2014-07-07

**Authors:** Yoshinori Mizokami, Takeshi Terao, Koji Hatano, Nobuhiko Hoaki, Kentaro Kohno, Yasuo Araki, Kensuke Kodama, Mayu Makino, Toshihiko Izumi, Tsuyoshi Shimomura, Minoru Fujiki, Takanori Kochiyama

**Affiliations:** ^1^Department of Neuropsychiatry, Faculty of Medicine, Oita UniversityOita, Japan; ^2^Department of Neurosurgery, Faculty of Medicine, Oita UniversityOita, Japan; ^3^ATR Promotions, Brain Activity Imaging CenterKyoto, Japan

**Keywords:** functional magnetic resonance imaging (fMRI), appreciation, beauty, oil painting, lingual gyrus, cuneus

## Abstract

Several studies have investigated neural correlates of aesthetic appreciation for paintings but to date the findings have been heterogeneous. This heterogeneity may be attributed to previous studies’ measurement of aesthetic appreciation of not only the beauty of paintings but also the beauty of motifs of the paintings. In order to better elucidate the beauty of paintings, it seems necessary to compare aesthetic appreciation of paintings and photographic analogs which included corresponding real images. We prepared for famous painters’ pictures and their photographic analogs which were set up to resemble each painting in order to investigate the hypothesis that there exist specific neural correlates associated with the aesthetic appreciation for paintings. Forty-four subjects participated in functional magnetic resonance study which required comparisons of aesthetic appreciation of paintings of still life and landscape versus photographic analogs including corresponding real images of still life and landscape. Bilateral cuneus and the left lingual gyrus were activated in the comparison of aesthetic appreciation of paintings versus photographic analogs. In conclusion, the present findings suggest a possibility of the existence of specific neural correlates associated with the aesthetic appreciation for paintings and that bilateral cuneus and the left lingual gyrus may be involved.

## Introduction

Neuroaesthetics is a relatively young field within cognitive neuroscience, concerned with the neural underpinnings of aesthetic experience of beauty, particularly in visual art. Neuroscientific investigations have approached this area using imaging and neurophysiological techniques, such as functional magnetic resonance imaging (fMRI), magnetoencephalography (MEG) and electroencephalography (EEG), but the results produced so far are very heterogeneous (Cinzia and Vittorio, 2009). Brown et al. (2011) meta-analyzed 93 neuroimaging studies of aesthetic appraisal across four sensory modalities and showed that the most concordant area of activation across all four modalities is the right anterior insula, which reflects the “viscerality” of aesthetic perception. Although this meta-analysis revealed the activation of the right anterior insula, regarding the aesthetic appreciation of oil paintings, so far, the activation of the orbitofrontal cortex has been reported (Kirk, 2008; Kirk et al., 2009a,b; Ishizu and Zeki, 2011). Also, the activation of lingual gyrus has been shown (Kawabata and Zeki, 2004; Vartanian and Goel, 2004) while that of occipital gyri has been reported (Vartanian and Goel, 2004; Cupchik et al., 2009).

Such heterogeneity may be attributed to previous studies’ measurement of aesthetic appreciation of not only the beauty of paintings but also the beauty of motifs of the paintings, which is inevitably contained in the case of representational paintings. To elucidate the neural substrates involved in perceiving the beauty of representational paintings, it seems necessary to compare the beauty of the paintings of still life and landscape and that of real images (photographic analogs) which contain the same motifs as the paintings. Because the representational paintings with still life and landscape as their motifs can be reproduced faithfully as photographs by using the real materials or scenes, and hence both can evoke the same aesthetic feeling attributable to the beauty of motifs. Thus, contrasting these two conditions is expected to reveal the neural correlates specifically associated with the beauty of paintings with controlling the beauty of the motifs. To the best of our knowledge, Lacey et al. (2011) firstly compared paintings and photographic analogs where famous painters’ pictures and roughly resembling photographic analogs were used. In a different way, Vartanian and Goel (2004) have filtered each original painting to make a control condition such that the filtered pictures retained the overall form of the original paintings but lacked perceptual details, showing that the comparison of original paintings versus filtered paintings activated the right fusiform gyrus.

Hence, in the present study, we prepared for famous painters’ pictures and their photographic analogs which were set up to resemble each painting in order to investigate the hypothesis that there exist specific neural correlates associated with the aesthetic appreciation for paintings.

## Materials and methods

### Subjects

Forty-four healthy subjects participated in the experiment but 5 subjects revealed artifacts. Therefore, 39 subjects’ data (mean age = 27.5 ± 5.7, 17 female) were submitted to analysis. Twenty-nine subjects were not interested in painting by themselves whereas the other 10 subjects were interested. The mean frequency of visiting museum was 0.76 times per year in the 29 subjects and 2.0 in the 10 subjects, respectively. All participants were right-handed and had normal or corrected to normal vision. They gave written informed consent to participate in this study according to procedures approved by the ethical committee at Oita University Faculty of Medicine.

### Stimuli

**Figure 1:**
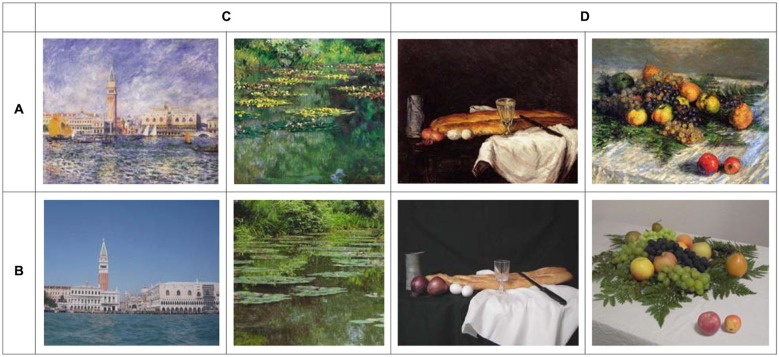
**Example of painting and photographic analogs stimuli in this study. (A)** Paintings **(B)** photographic analogs **(C)** landscape **(D)** still life.

Several days before imaging experiment, each subject slowly and carefully viewed 20 paintings of which contents were still life and land scape by famous painters such as Cezanne, Monet and so on. The 20 paintings were displayed on the table and ranked subjectively. Consequently, they were divided into 3 groups consisting of 5 paintings of the most beautiful, 10 paintings of more beautiful, and 5 paintings of not so beautiful by each subject. Thereafter, 5 paintings of the 10 paintings of more beautiful were randomly selected by the researcher (Y.M.). In sum, 15 paintings (5 of the most beautiful, 5 of more beautiful, and 5 of not so beautiful) were used for the following imaging experiment. Twenty photographs were taken in advance by the researcher (Y.M.) to imitate the 20 paintings as similarly as possible. Every effort was made to prepare similar motifs to imitate the corresponding paintings. Independently, the 20 photographic analogs were displayed on the table and ranked subjectively. These photographs (i.e., photographic analogs) were used as a control for the imaging experiment as shown in Figure 1. Since artistic paintings could not be used, the set of photographs of artistic paintings were used as Cela-Conde et al. (2009). It should be noted that the content of paintings and photographs were not the same across subjects and depended on individual subject’s aesthetic appreciation of the paintings. Moreover, there was no significant difference in luminance between paintings and photographs.

### Experimental procedure

**Figure 2:**
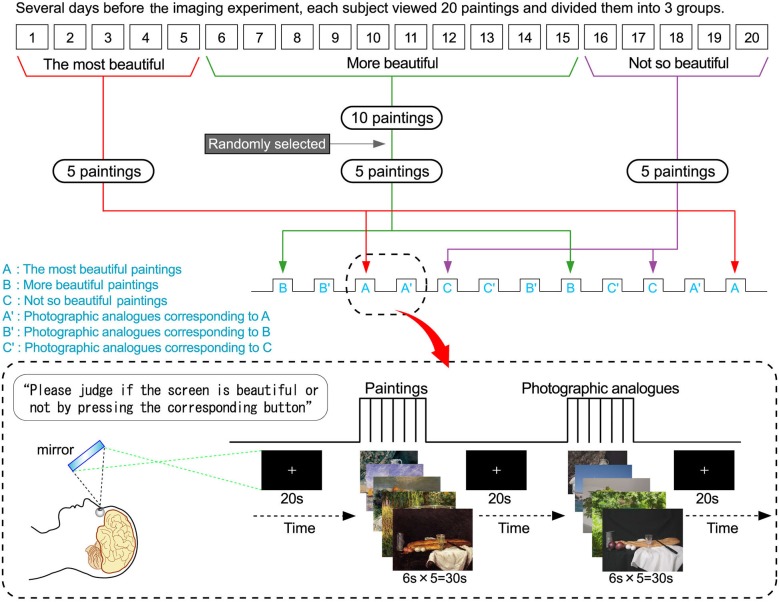
**Protocol**. Several days before imaging experiment, each subject slowly and carefully viewed 20 paintings which were still life and landscape by famous painters such as Cezanne, Monet and so on. They were divided into 3 groups consisting of 5 paintings of the most beautiful, 10 paintings of more beautiful, and 5 paintings of not so beautiful by each subject. Thereafter, 5 paintings of the 10 paintings of more beautiful were randomly selected by the researcher. This study was block-design and all blocks had a sequence of 5 screens which consisted of paintings or photographic analogs. In sum, 15 paintings (5 of the most beautiful, 5 of more beautiful, and 5 of not so beautiful) were used for the following imaging experiment. Three painting blocks were presented to the subjects comprising the most beautiful (block A), more beautiful (block B) and not so beautiful (block C) while another 3 photographic analog blocks labeled A’, B’, and C’ were presented that corresponded to (or imitated) block A, B, and C, respectively. These photographs (i.e., photographic analogs) were used as a control for the imaging experiment. Prior to each block, a fixation cross was presented for 20 s. The sequence of the presentation of the blocks was B, B’, A, A’, C, C’, B’, B, C’, C, A’, and A. Subjects were instructed as follows: “Please judge if the screen is beautiful or not by pressing the corresponding button”.

This study was block-design and all blocks had a sequence of 5 screens which consisted of paintings or photographic analogs. As shown in Figure 2, several experimental conditions/blocks were presented. Three painting blocks were presented to the subjects comprising the most beautiful (block A), more beautiful (block B) and not so beautiful (block C) while another 3 photographic analog blocks labeled A’, B’, and C’ were presented that corresponded to (or imitated) block A, B, and C, respectively. These visual stimuli were presented using Presentation (version 14.1) and projected via a forward projection system onto a translucent screen placed at the end of the magnet’s gurney. Subjects viewed the screen through a mirror attached to the head coil. Prior to each block, a fixation cross was presented for 20 s. The sequence of the presentation of the blocks was B, B’, A, A’, C, C’, B’, B, C’, C, A’, and A. Subjects were instructed as follows: “Please judge if the screen is beautiful or not by pressing the corresponding button”. It should be noted that there was no significant difference in luminance between paintings and photographs.

### MRI

Functional MR images were collected using Siemens magnetom verio 3T MRI system. A time course series of 174 volumes was acquired with a T2-weighted single shot gradient echo planar imaging (EPI) sequence. Each volume consisted of 36 slices, with a slice thickness of 3 mm and a gap of 0.75 mm, and covered the almost the whole brain. Images were acquired in the axial plane (TR = 3000 ms; TE = 30 ms; FOV = 210 mm; voxel size = 3 × 3 × 3 mm). The total acquisition time was 10 min 8 s, including periods for signal equilibration. T1-weighted structural images were acquired with 3-D magnetization prepared rapid gradient echo (MPRAGE) in the sagittal plane (TR = 2040 ms; TE = 2.53 ms; TI = 900 ms; the flip angle was 9°; FOV = 192 mm; voxel size = 1 × 1 × 1 mm).

### fMRI data analysis

All fMRI analysis was performed in SPM8 (Statistical Parametric Mapping software, University College of London, London, UK).^1^ Preprocessing (movement correction, normalization to the MNI EPI template, smoothing with an isotropic 8 mm FWHM kernel, and resampling to 2 mm cubic voxels) were performed first. Each individual data set was carefully screened for data quality via inspection for image artifacts and excessive head motion (>3 mm head motion or 2° head rotation).

In the first level analyses, we used following parameters: Interscan Interval 3 s, Microscan Resolution 36, and Microtime Onset 18. These values followed setting of MR acquisition. Each condition was modeled with a boxcar function and convoluted with a canonical hemodynamic response function. Low frequency drifts were removed using a temporal high-pass filter with a cutoff of 128 s. Serial autocorrelation was also corrected using AR (1) model. We created 6 beta images in each subject: most beautiful paintings, more beautiful paintings, not so beautiful paintings, most beautiful photographic analogs, more beautiful photographic analogs, not so beautiful photographic analogs.

The second level (random-effects) analyses using the full factorial module in SPM8. We performed analysis of variance (ANOVA) using visual sources (paintings and photographic analogs) and rating (most beautiful, more beautiful and not so beautiful) as factors using 6 beta images created in the first level. Statistical thresholds for full factorial analysis were set at *p* < 0.001 at voxel level (uncorrected), *p* < 0.05 at cluster level (family-wise error (FWE) corrected).

## Results

During the fMRI experiment, subjects appreciated whether the painting or photographic analog was beautiful or not, again. In comparison with the pre-experimental appreciation, 83.6% of (pre-appreciated) the most beautiful paintings were appreciated as beautiful. Similarly, 64.1% of more beautiful paintings and 33.3% of not so beautiful paintings were appreciated as beautiful. Regarding photographic analogs, 68.9% of photographic analogs to the most beautiful paintings, 59.7% of photographic analogs to more beautiful paintings, and 39.7% of photographic analogs to not so beautiful paintings were appreciated as beautiful. There was no significant difference in the number of appreciation of beautiful between all paintings and all photographic analogs (χ^2^ = 1.86, *p* = 0.40). During the experiment appreciation, subjects appreciated the painting as beautiful (58.7%) and they appreciated the photographic analogs as beautiful (57.5%) with no significant difference. Nonetheless, the association between the pre-experimental appreciation and the experimental appreciation was significantly positive for both paintings and photographic analogs in all 39 subjects. Therefore, it seems likely that there was a substantially difference in the aesthetic impact (paintings > photographic analogs), but the way of question (beautiful or not in the experiment) could not draw the difference. In order to investigate this possibility, we compared the rankings of the most beautiful paintings and those of their corresponding photographic analogs. In the ranking method, “1” is the most beautiful and a lower ranking value means more beautiful. As a result, the mean of the rankings of the most beautiful paintings was 3.0 (SD = 1.4) and that of those of their corresponding photographic analogs was 6.9 (SD = 5.1) (*t* = −11.8, *p* < 0.0001). Therefore, there was a significant difference in the aesthetic impact (paintings > photographic analogs) in the pre-experimental setting.

**Figure 3:**
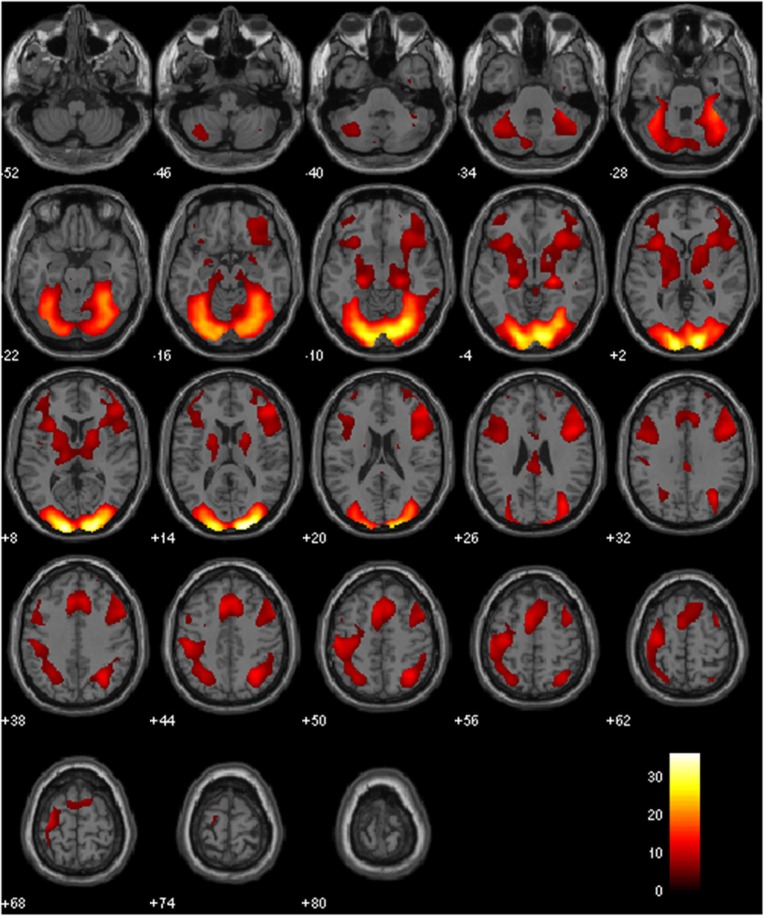
**Various regions including bilateral cingulate gyrus and bilateral medial frontal gyrus were activated.** The statistical significance refers to *p* < 0.05 (FWE corrected) at voxel level.

**Figure 4:**
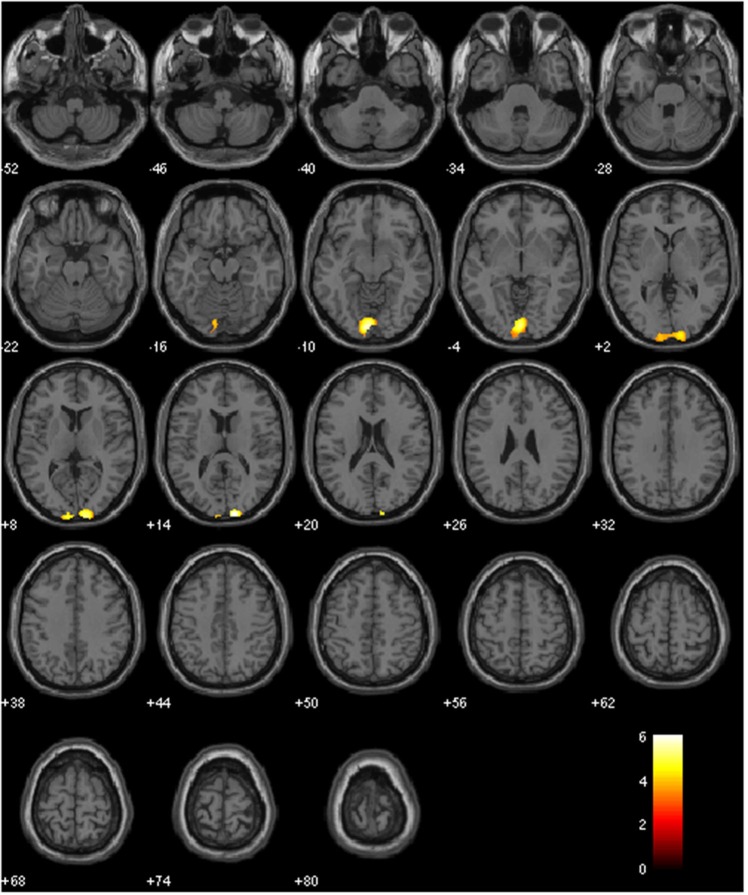
**Activations in the contrast: aesthetic appreciation of all paintings > corresponding all photographic analogs**. The comparison of aesthetic appreciation of 15 paintings versus corresponding 15 photographic analogs revealed activations in bilateral cuneus and left lingual gyrus, The statistical significance refers to *p* < 0.001 (uncorrected) at voxel level, *p* < 0.05 at cluster level (FWE corrected).

Table 1 and Figure 3 show that various regions including bilateral cingulate gyrus and bilateral medial frontal gyrus were activated. The results of ANOVA were shown in Figure 4 respectively. Activated regions in both positive effect of painting and positive effect of photographic analogs were described in Table 2. The positive effect of aesthetic appreciation of all three painting contrasts versus corresponding all three photographic analog contrasts revealed activations in bilateral cuneus and left lingual gyrus. On the other hand, positive effect of aesthetic appreciation of all three photographic analog contrasts versus all three painting contrasts revealed other regions shown in Table 1. There was no significant association between the brain responses and subjective ratings.

**Table 1:** **Brain regions and coordinates of clusters and local maxima for aesthetic appreciation of paintings**.

	**Brodmann Area**	**MNI coordinates**				
**Talairach regions**		***x***	***y***	***z***	**Cluster size in voxels**	***Z*-score**
Right Middle Occipital Gyrus	19	30	−96	14	43922	Inf
Right Lingual Gyrus	N. A.	8	−92	−6		Inf
Left Lingual Gyrus	N. A.	−6	−92	−12		Inf
Right Medial Frontal Gyrus	6	6	28	42	8168	Inf
Left Medial Frontal Gyrus	8	−4	16	48		Inf
Left Cingulate Gyrus	32	−10	22	42		Inf
Right Cingulate Gyrus	N. A.	2	−30	28	681	6.38
Right Cerebellar Tonsil	N. A.	34	−60	−50	96	5.37
Right Inferior Semi-Lunar Lobule	N. A.	30	−68	−48		5.32
Right Middle Frontal Gyrus	N. A.	28	48	14	61	5.28
Right Middle Temporal Gyrus	N. A.	48	−26	−6	43	5.27
Left Uncus	N. A.	−34	−6	−36	21	5.27
Right Middle Temporal Gyrus	21	64	−36	−8	58	5.17
Left Cerebellar Tonsil	N. A.	−18	−36	−50	13	5.11
Right Inferior Semi-Lunar Lobule	N. A.	14	−64	−50	6	4.84
Left Middle Frontal Gyrus	8	−46	24	44	1	4.67

*Coordinates of the maximal point of activation and the associated z-values are shown. The activations are significant at p < 0.05 (FWE corrected) at voxel level*.

**Table 2:** **Brain regions and coordinates of clusters and local maxima for positive effect of aesthetic appreciation of paintings versus photographic analogues**.

	**Brodmann Area**	**MNI coordinates**				
**Talairach regions**		***x***	***y***	***z***	**Cluster size in voxels**	***Z*-score**
Right Cuneus	18	14	−100	14	1628	5.87
Left Lingual Gyrus	18	−2	−88	−8		5.66
Left Cuneus	18	−8	−102	8		4.80

*Coordinates of the maximal point of activation and the associated z-values are shown. The activations are significant at p < 0.001 (uncorrected) at voxel level, p < 0.05 at cluster level (FEW corrected)*.

## Discussion

As aforementioned, perceived beauty of representational paintings may consist of the beauty of the motifs of the paintings plus the beauty of the paintings themselves, although, strictly speaking, the beauty may be modified by the use of the set of photographs of the artistic paintings for the presentation in fMRI. On the other hand, perceived beauty of photographic analogs may consist of the beauty of the motifs of the corresponding paintings plus the beauty of the photographs themselves. The present study could not measure each part, but perceived beauty of the paintings minus perceived beauty of the photographic analogs could have cancelled the beauty of the motifs and thereby estimated the beauty of the paintings themselves although the motifs of the photographic analogs were not completely the same as those of the paintings. In any case, the rate of beautiful appreciation was clearly decreased in line with the decrease of pre-appreciated beauty, indicating the validity of the present method to assess the appreciation of beauty of paintings.

Although during the experiment appreciation subjects appreciated the painting as beautiful (58.7%) and they appreciated the photographic analogs as beautiful (57.5%) with no significant difference, it seems likely that there was a substantially difference in the aesthetic impact (paintings > photographic analogs), but the way of question (beautiful or not in the experiment) could not draw the difference. In order to investigate this possibility, we compared the rankings of the most beautiful paintings and those of their corresponding photographic analogs. As a result, the mean of the rankings of the most beautiful paintings was 3.0 (SD = 1.4) and that of those of their corresponding photographic analogs was 6.9 (SD = 5.1) (*t* = −11.8, *p* < 0.0001). Therefore, there was a significant difference in the aesthetic impact (paintings > photographic analogs) in the pre-experimental setting. Taking the significantly positive association between the pre-experimental appreciation and the experimental appreciation, it seems likely that there was a significant difference in the aesthetic impact (paintings > photographic analogs) in the experimental setting.

Again, the purpose of the present study was to investigate the hypothesis that there exist specific neural correlates associated with the aesthetic appreciation for paintings. Therefore, we focused the comparison of aesthetic appreciation of paintings versus photographic analogs as a control. With regard to the comparison of aesthetic appreciation of 15 paintings versus 15 photographic analogs, bilateral cuneus and the left lingual gyrus were activated. These findings suggest that there exist specific neural correlates associated with the aesthetic appreciation for paintings and that they may be associated with bilateral cuneus and the left lingual gyrus.

Kawabata and Zeki (2004) showed that during aesthetical appreciation, paintings of landscapes produced activation in the anterior part of lingual gyrus, which is at least partly in accordance with our findings which revealed activation in the left lingual gyrus. Cupchik et al. (2009) revealed that the comparison of aesthetic appreciation (viewing representational paintings with aesthetic appreciation) versus baseline (viewing non-representational paintings without aesthetic or pragmatic appreciation) activated the bilateral insula (BA13) and bilateral occipital gyri (BA18/19) while the comparison of pragmatic appreciation versus baseline activated the right fusiform gyrus and bilateral occipital gyri (BA19) which is at least partly in accordance with our findings which revealed activation in bilateral cuneus (BA18) and the left lingual gyrus (BA18). Taken together, these studies suggest that bilateral cuneus and the left lingual gyrus may be neural correlates closely associated with aesthetic appreciation of paintings, which support our findings. With regard to the function of lingual gyrus, Chatterjee et al. (2009) argued that activation in this region represents its sensitivity to beauty, which is in line with the present findings.

Huang et al. (2011) proposed that the activation of brain networks including frontopolar cortex and right precuneus rather than a single cortical area in their paradigm supports the art scholar’s view that esthetic judgments are multi-faceted and multi-dimensional in nature. The present findings also suggest that in our paradigm perceived beauty of the artistic paintings may be associated with the brain network which may include various regions including bilateral cingulate gyrus and bilateral medial frontal gyrus (Figure 3 and Table 1) and specifically bilateral cuneus and left lingual gyrus (Figure 4 and Table 2) were activated.

One limitation is that the motifs were limited to still life and landscape of French late 19th century painters and excluded abstract paintings. Another limitation is methodological which contains a small number of blocks and order effects where paintings are always presented first. Moreover, it is unknown whether the block design is the best method. Also, aesthetic impact is not a unitary factor, being generated by fear, disgust, alarm as much as beauty or tranquility and these factors should have been considered as covariates in the analysis of our paradigm. Finally, the activation in what is termed the cuneus and lingual gyrus (Figure 4) corresponds to the foveal representation of the early visual areas, and may be interpreted as the expected fine-grain difference between the textural quality of the paintings versus the photographs. This textural difference cannot be completely differentiated from aesthetic. Further studied are required to generalize the present findings.

In conclusion, the present findings suggest a possibility that bilateral cuneus and the left lingual gyrus may be also closely associated with aesthetic appreciation of representational paintings.

## Conflict of interest statement

The authors declare that the research was conducted in the absence of any commercial or financial relationships that could be construed as a potential conflict of interest.

## Footnotes

^1^http://www.fil.ion.ucl.ac.uk/spm/
